# rTMS Regulates the Balance Between Proliferation and Apoptosis of Spinal Cord Derived Neural Stem/Progenitor Cells

**DOI:** 10.3389/fncel.2019.00584

**Published:** 2020-01-28

**Authors:** Chen-Guang Zhao, Jie Qin, Wei Sun, Fen Ju, Yong-Lin Zhao, Rui Wang, Xiao-Long Sun, Xiang Mou, Hua Yuan

**Affiliations:** ^1^Department of Rehabilitation Medicine, Xijing Hospital, Fourth Military Medical University, Xi’an, China; ^2^Department of Orthopedics, The Second Affiliated Hospital of Xi’an Jiaotong University, Xi’an, China; ^3^Department of Oncology, The Second Affiliated Hospital of Xi’an Jiaotong University, Xi’an, China

**Keywords:** rTMS, proliferation, apoptosis, neural stem/progenitor cells, BDNF

## Abstract

Repetitive transcranial magnetic stimulation (rTMS) is a noninvasive technique that uses electromagnetic fields to stimulate the brain. rTMS can restore an impaired central nervous system and promote proliferation of neural stem/progenitor cells (NSPCs), but optimal stimulus parameters and mechanisms underlying these effects remain elusive. The purpose of this study is to investigate the effect of different rTMS stimulus parameters on proliferation and apoptosis of spinal cord-derived NSPCs, the expression of brain-derived neurotrophic factor (BDNF) after rTMS, and the potentially underlying pathways. NSPCs were isolated from mice spinal cord and stimulated by different frequencies (1/10/20 Hz), intensities (0.87/1.24/1.58 T), and number of pulses (400/800/1,500/3,000) once a day for five consecutive days. NSPC proliferation was analyzed by measuring the neurosphere diameter and Brdu staining, apoptosis was detected by cell death enzyme-linked immunosorbent assay (ELISA) and flow cytometry, and NSPC viability was assessed by cell counting kit-8 assay. We found that specific parameters of frequency (1/10/20 Hz), intensity (1.24/1.58 T), and number of pulses (800/1,500/3,000) promote proliferation and apoptosis (*p* < 0.05 for all), but 20 Hz, 1.58 T, and 1,500 pulses achieved the optimal response for the NSPC viability. In addition, rTMS significantly promoted the expression of *BDNF* at the mRNA and protein level, while also increasing Akt phosphorylation (Thr308 and Ser473; *p* < 0.05). Overall, we identified the most appropriate rTMS parameters for further studies on NSPCs *in vitro* and *in vivo*. Furthermore, the effect of magnetic stimulation on NSPC proliferation might be correlated to BDNF/Akt signaling pathway.

## Introduction

Repetitive transcranial magnetic stimulation (rTMS) is a widely used noninvasive technique that uses electromagnetic fields to stimulate the brain. Generated magnetic fields penetrate the skull with minimal attenuation and produce electrical currents in cortical layers. rTMS has a direct effect on the excitability of regions functionally connected with cortical and subcortical structures. The result of rTMS is affected by the intensity and frequency of the stimulation: low frequency rTMS (≤1 Hz) and high frequency rTMS (≥5 Hz) decrease and increase the cortical excitability, respectively (Ridding and Ziemann, 2010).

rTMS has been reported to treat many neurological diseases including traumatic brain injury, spinal cord injury (SCI), neurodegenerative disorders, schizophrenia, and substance addiction (Tazoe and Perez, 2015; Benussi et al., 2018; Shin et al., 2018). The therapeutic effect induced by rTMS might be associated to several processes, such as increased neural stem and progenitor cell growth and neuronal differentiation, release of neurotrophic factors (e.g., brain-derived neurotrophic factor, BDNF), and regulation of neuronal excitability and cerebral blood flow (Lee et al., 2015). However, the exact mechanism underlying rTMS function is still elusive and needs to be further investigated.

The discovery of resident neural stem cells in the adult central nervous system, especially in the spinal cord (Barnabé-Heider and Frisén, 2008), has evoked interest in transplantation therapies of SCI based on the recruitment of neural stem/progenitor cells (NSPCs; Sabelström et al., 2014). Numerous techniques were explored to promote the proliferation of NSPCs, including rTMS, but the most appropriate parameters were not clear (Guo et al., 2014).

In this study, we aimed to explore the effect of rTMS at different intensity, frequency, and number of pulses on spinal cord-derived NSPC apoptosis and proliferation, shedding light on potential underlying mechanisms.

## Materials and Methods

### Spinal Cord-Derived NSPC Isolation, Culture, and Differentiation

The study was approved by the Medical Ethics Committee of the Xijing Hospital of the Fourth Military Medical University (No.XJYYLL-2015134) and was conducted according to the ARRIVE guidelines and National Institutes of Health Guide for the Care and Use of Laboratory Animals. NSPCs were isolated from spinal cords of 8-weeks-old adult C57/BL6 mice, according to our previously published method (Wang et al., 2018). Briefly, thoracic spinal cords were isolated, triturated, and passed through a 70-μm strainer. Then, cells were cultured in NSPC growth media (DMEM/F12, 11320033, B-27, 12587010, N-2, 17502048, Gibco) according to the manufacturer’s instructions. Cells were passaged every 6–8 days and experiments were conducted between the third and the seventh passage. For adherent cultures, cells were harvested and seeded on slips coated with poly-L-lysine (0.01%, P4707, Sigma–Aldrich, St. Louis, MO, USA). For NSPC differentiation specific differentiation media were used replacing the NSPC growth medium according to the Gibco neurobiology protocol handbook, as we previously described (Wang et al., 2018).

### *In vitro* Repetitive Magnetic Stimulation

**Figure 1:**
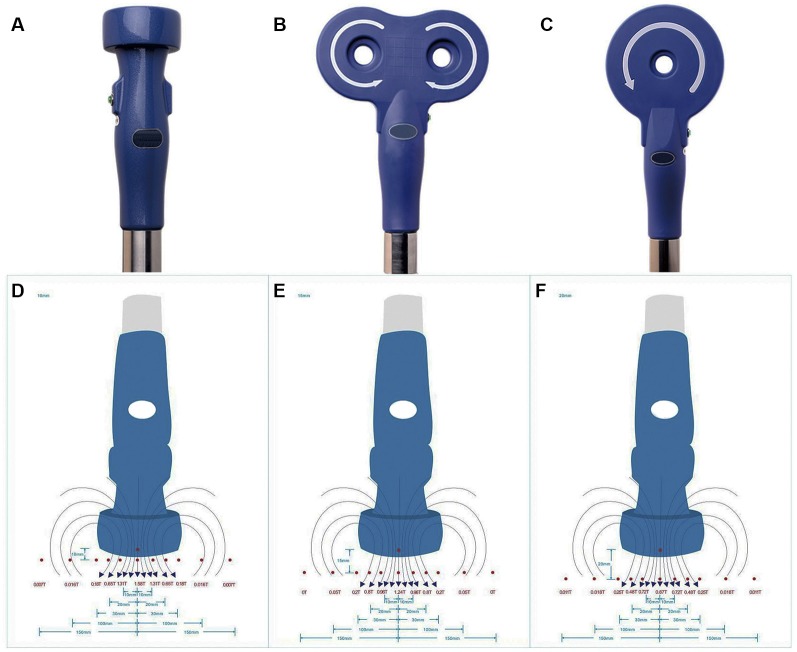
Appearance of stimulation coil and intensities of repetitive transcranial magnetic stimulation (rTMS). Appearance of experimental coil **(A)**, figure-8 coil **(B)** and circular coil **(C)**. Schematic diagram of different intensities of rTMS were measured at 1.0 cm **(D)**, 1.5 cm **(E)** and 2.0 cm **(F)** from the center of the coil to the bottom of the culture dish.

rTMS was applied on NSPCs with a CCY-1 stimulator (YIRUIDE Medical Equipment Company, China) using the experimental coil as recommended (Figure 1A), easily distinguishable from figure-8 coil (Figure 1B) or circular coil (Figure 1C). Before stimulation, the intensity at different distances (from the center of the coil to the bottom of the culture dish) was measured, and the exact intensity of the magnetic field was recorded as schematized in Figure 1. For detecting the effects of different stimulus frequencies, the parameters were set at 1/10/20 Hz of stimulation with a total of 1,500 pulses (peak value 1.58 T) once a day for five consecutive days. For detecting the effects of different intensities of the magnetic field, we tested 0.87/1.24/1.58 T (1.0/1.5/2.0 cm distance from the center of the coil to the bottom of the culture dish) of 20-Hz stimulation with a total of 1,500 pulses once a day for five consecutive days. The effects of different stimuli pulses were evaluated by setting the parameters at 400/800/1,500/3,000 pulses of 20-Hz stimulation (peak value 1.58 T) once a day for five consecutive days. For the control group, NSPC plates were placed under the experimental coil at the same room temperature but were subjected to no stimulation. The first treatment was commenced 8 h after NSPC plating, and the subsequent treatments were commenced every morning since the 2nd day for 4 days. All the experiments were performed in three biological replicates.

### Quantitative Real-Time Reverse Transcription-PCR (qRT-PCR)

Total RNA was isolated using TRIzol reagent (15596018, Invitrogen, Waltham, MA, USA) according to the manufacturer’s instructions. First-strand cDNAs were synthesized by using the PrimeScript RT Reagent Kit with gDNA Eraser (RR047A, TaKaRa) according to the manufacturer’s instructions. To quantify the mRNA expression of *BDNF*, qRT-PCR using TB Green Premix Ex Taq II (RR820A, TaKaRa) was performed. Primer pairs were as follows—BDNF: AATCCCATGGGTTACACGAA (forward) and AAGTTGTGCGCA AATGACTG (reverse); GAPDH: CCTCTGGAAAGCTGTGGCGT (forward) and TTGGAGGCCATGTAGGCCAT (reverse). The reaction conditions were as follows: 95°C for 30 s, 40 cycles at 95°C for 5 s, and 60°C for 30 s. The relative expression was quantified using the 2^−ΔΔct^ method. *BDNF* mRNA levels were then expressed as fold changes after normalization to GAPDH levels.

### Western Blot Analysis

NSPCs were harvested and homogenized with a radio immunoprecipitation assay (RIPA) lysis buffer (89900, Thermo Fisher Scientific, Waltham, MA, USA) containing a protease inhibitor cocktail (11697498001, Roche, Basel, Switzerland). The samples were centrifuged at 12,000 rpm at 4°C, supernatants were collected, and protein content was determined by NanoDrop spectrophotometer (ND-1000, Thermo Fisher Scientific, Waltham, MA, USA). Equal amounts of total protein (20 μg) were separated onto 10% sodium dodecyl sulfate-polyacrylamide gel electrophoresis (SDS-PAGE), transferred to polyvinylidene fluoride (PVDF) membranes, and blocked with 5% skim milk in Tris buffered saline plus Tween (TBST) buffer at room temperature. Membranes were then incubated overnight at 4°C with the following primary antibodies: anti-phospho-Akt (Thr308; 1:500, #13038 Cell Signalling Technology, Danvers, MA, USA), anti-phospho-Akt (Ser473; 1:500, #4060, Cell Signalling Technology, Danvers, MA, USA), anti-Akt (1:1,000, #4685, Cell Signalling Technology, Danvers, MA, USA), anti-BDNF (1:500, ab108319, Abcam, Cambridge, UK), and anti-β-actin (1:1,000, #4970, Cell Signalling Technology, Danvers, MA, USA). Membranes were washed with TBS and incubated with horseradish peroxidase-conjugated secondary antibody (1:2,000, ab205718, Abcam, Cambridge, UK) for 2 h. Protein bands were detected by exposure to X-ray films. Films were scanned, and digital images were analyzed using the ImageJ software (version 1.45, NIH, USA). Relative protein levels were expressed as fold changes after normalization to β-actin.

### Immunofluorescence Study

Cell cultures were fixed with 4% cold paraformaldehyde (PFA) for 5 min at room temperature. After washing, the primary antibody solution containing anti-nestin (1:200, MAB353, Chemicon), anti-TUJ1 (1:100, MAB1637, Chemicon), anti-glial fibrillary acidic protein (GFAP; 1:400, Z033401–2, Dako), anti-oligodendrocyte marker O4 (1:25, MAB345, Chemicon), and anti-TrkB (1:200, ab18987, Abcam, Cambridge, UK) was added to each coverslip and incubated overnight at 4°C. The primary antibody was removed and samples were incubated with the corresponding secondary antibody solution (1:500, A-10680, A-11030, A-11081, A-11035, Thermo Fisher Scientific, Waltham, MA, USA) and 4’,6-diamidino-2-phenylindole (DAPI; 1:1,000, ab228549, Abcam, Cambridge, UK) for 45 min at room temperature. Finally, samples were washed with phosphate buffered saline (PBS) and observed using a fluorescent microscope (Zeiss Axio Vert. A1).

### Neurosphere Diameter Measurement

Neurosphere diameter was measured in three biological replicates, and for each repetition, 10 micrographs were randomly captured under 200× magnification from culture dish after the final rTMS stimulation. Then the average diameter of neurospheres was calculated from the 30 micrographs.

### BrdU Incorporation

Cells were plated on coverslips and incubated with 10 μM BrdU (B5002, Sigma–Aldrich, St. Louis, MO, USA) for 8 h at 37°C. Cells were then fixed with 4% PFA and incubated with 2 mol/L of HCl, followed by 1 mol/L Na_2_B_4_O_7_. After washing, cells were incubated with anti-BrdU antibody (1:250, ab6326, Abcam, Cambridge, UK) for 4 h, goat antirat Alexa Fluor 555-conjugated secondary antibody for 1 h, and Hoechst stain for 5 min at room temperature. Finally, cells were examined with a fluorescence microscope (Zeiss Axio Vert. A1). Data were expressed as the number of BrdU-positive cells/total number of cells. The final percentage of positive cells was calculated from three biological replicates. In each biological repetition, 10 micrographs were randomly captured from three cover slips.

### Flow Cytometry Assay

To detect cell apoptosis, flow cytometry was performed using the Annexin V-fluorescein isothiocyanate (FITC) and propidium iodide (PI) double staining kit (556547, BD Biosciences, San Jose, CA, USA) according to the manufacturer’s instructions. Briefly, after magnetic stimulation, NSPCs were collected and resuspended in a 500-μl binding buffer. Then, aliquots (100 μl) of the solution were transferred for incubation with 5-μl FITC-Annexin V and 5-μl PI, followed by gently vortexing at room temperature avoiding light for 10 min. Samples were analyzed using a Beckman Epics XL-MCL flow cytometer within 1 h. Flow cytometry assays were tested three times in each biological experiment, and averages of a total of three biological replicates were used for statistical analysis.

### Cell Death Assay

The cell death detection enzyme-linked immunosorbent assay (ELISA) Plus kit (11544675001, Roche, Basel, Switzerland) was used to measure the relative amounts of mono- and oligonucleosomes released from the apoptotic cells in the cytoplasmic fraction. The ELISA was performed following the manufacturer’s instructions. Briefly, cells were collected and homogenized in 400 μl of incubation buffer. After the incubation, the cell lysates containing histone/DNA components were obtained. Then, antihistone and anti-DNA-peroxidase antibodies were added followed by the peroxidase substrate. Finally, the absorbance was recorded at 405 nm with a microplate reader (ELx808, BioTek Instruments Inc., Winooski, VT, USA). ELISAs were tested three times in each biological repetition; averages of a total of three biological replicates were used for statistical analysis.

### Cell Counting Kit-8 (CCK-8) Assay

CCK-8 assay, which is considered as a sensitive, nonradioactive colorimetric assay, was used to determine the number of viable cells. Briefly, NSPCs were cultured in NSPC growth media for 72 h, and CCK-8 solution (CK04, Dojindo) was added at a final concentration of 10 μl/100 μl and incubated for 4 h at 37°C. Optical density (OD) at 450 nm was measured with a microplate reader (ELx808, BioTek Instruments Inc., Winooski, VT, USA). CCK-8 levels were tested six times in one biological repetition, and averages of a total of three biological replicates were used for final analysis.

### BDNF ELISA

The concentration of BDNF in supernatants from NSPCs after rTMS was determined by an ELISA kit (MU30097, Bio-Swamp) in accordance with the manufacturer’s guidelines. BDNF levels were determined by reading the OD of each sample at the wavelength of 450 nm and calculating the standard curve. ELISAs were tested three times in one biological repetition, and averages of a total of three biological replicates were used for statistical analysis.

### Statistical Analysis

All data were expressed as mean ± SD and analyzed using SPSS 20.0 (SPSS, Chicago, IL, USA) software. Significant differences between two groups were evaluated by the student’s *t*-test; analysis of variance (ANOVA) was used for comparison in more than two groups, followed by least significant difference (LSD) to conduct a *post hoc* test. The level of significance for all comparisons was set at *p* < 0.05.

## Results

### Culture and Identification of NSPCs

**Figure 2:**
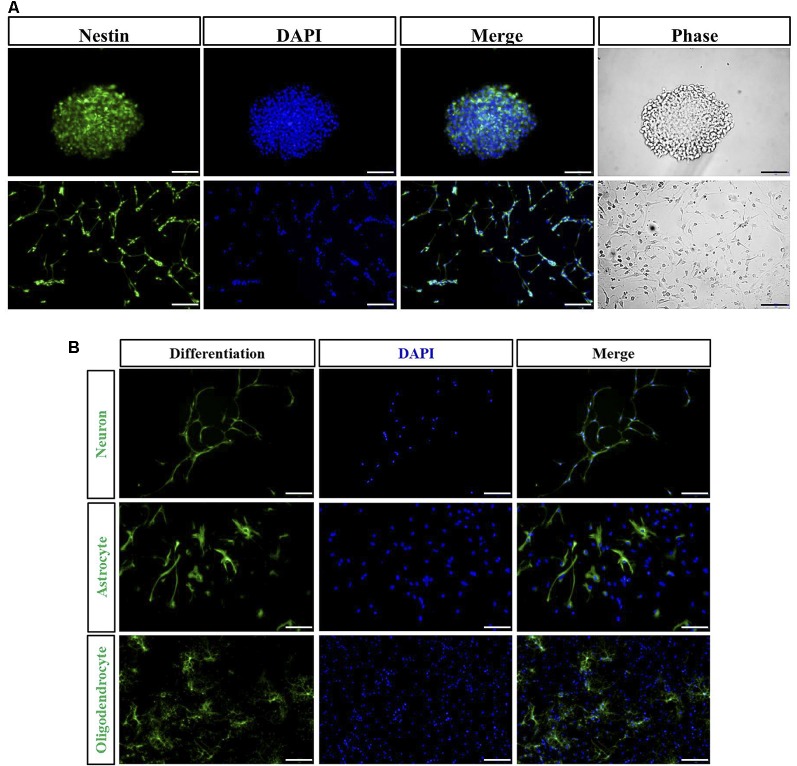
Immunocharacterization and differentiation of neural stem/progenitor cells (NSPCs). **(A)** NSPCs isolated from spinal cord under suspension (Bar = 50 μm) and adherent culture (Bar = 100 μm) were identified by Nestin (green), DAPI (blue), and the phase contrast images. **(B)** NSPCs were capable of differentiating into neurons (TUJ-1 positive), astrocytes (GFAP positive), and oligodendrocytes (O4 positive). Cell nuclei were counterstained with DAPI (blue). Bar = 100 μm.

The majority of the cells isolated from spinal cord gradually adhered to the wall after 1 day. Cells were found to grow into neurospheres after 2 days. Nestin positive cells detected by immunofluorescence were both found in floating neurospheres and adherent NSPCs (Figure 2A). Following the addition to NSPCs of specific differentiation medium, neurospheres were capable of developing into heterogeneous populations of neurons, astrocytes, and oligodendrocytes. TUJ-1-positive neuron (8.9 ± 1.4%), GFAP-positive astrocyte (65.1 ± 3.5%), and O4-positive oligodendrocyte (12.4 ± 1.8%) were observed by immunofluorescence after 10 days of incubation (Figure 2B).

### Different Frequencies of rTMS on NSPC Proliferation and Apoptosis

**Figure 3:**
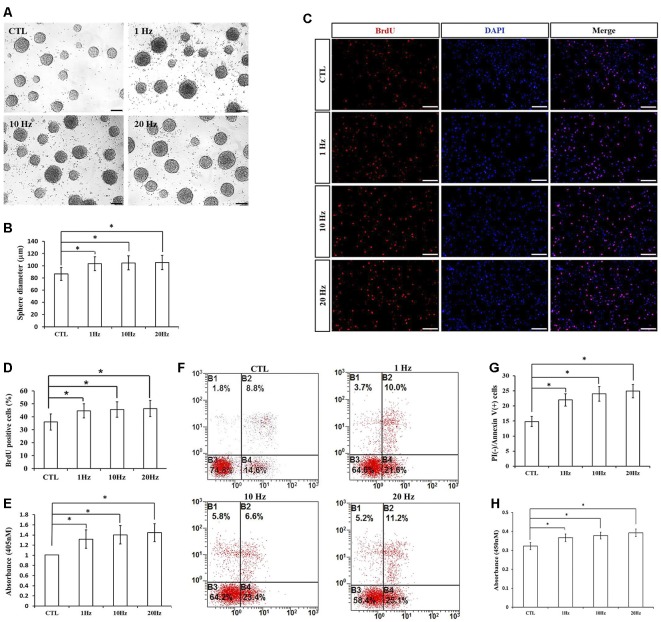
Effects of different frequencies of rTMS on NSPC proliferation and apoptosis. **(A)** Neurospheres in CTL and rTMS group (*n* = 3). **(B)** Neurosphere diameter increased in rTMS groups (1, 10, and 20 Hz; *n* = 3). **(C)** BrdU positive NSPCs in CTL and rTMS groups (*n* = 3). **(D)** BrdU positive rate increased in rTMS groups (1, 10, and 20 Hz; *n* = 3). **(E)** Apoptosis rate increased in rTMS groups (1, 10, and 20 Hz) assessed by cell death enzyme-linked immunosorbent assay (ELISA; *n* = 3). **(F)** Apoptosis rate in CTL and rTMS groups assessed by flow cytometry analysis (*n* = 3). **(G)** Apoptosis rate increased in rTMS group (1, 10, and 20 Hz; *n* = 3). **(H)** Cell viability increased in the rTMS group (1, 10, and 20 Hz; *n* = 3). *p < 0.05 compared with the control group. Bar = 100 μm.

To explore the effects of different frequencies of rTMS on NSPCs, we assayed different parameters (1, 10, and 20 Hz) while the intensity and the number of pulses were set to 1.58 T and 1,500, respectively. We observed that compared to the control group, rTMS (1, 10, and 20 Hz) stimulated NSPC proliferation (*p* < 0.05), as suggested by evaluating the neurosphere diameter (Figures 3A,B) and BrdU positive cell rate (Figures 3C,D). Different rTMS frequencies also increased the apoptosis rate of NSPCs (*p* < 0.05), as showed by the apoptosis ELISA analysis (Figure 3E) and flow cytometry results (Figures 3F,G). CCK-8 assay showed that all different frequencies significantly increased cell viability compared with control (*p* < 0.05; Figure 3H), but no statistical differences were observed in proliferation, apoptosis, or cell viability between the three stimulation groups (*p* > 0.05). All these results indicated that different frequencies (1, 10, and 20 Hz) promoted NSPC proliferation and increase cell viability.

### Different Intensities of rTMS on NSPC Proliferation and Apoptosis

**Figure 4:**
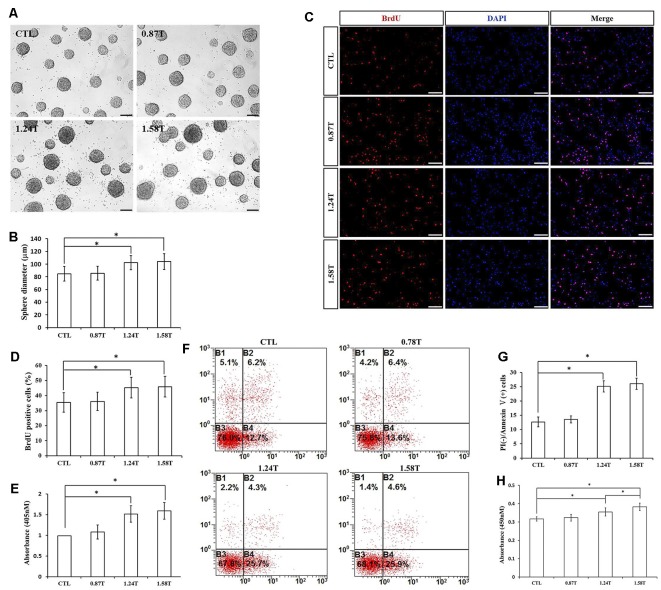
Effects of different intensities of rTMS on NSPC proliferation and apoptosis. **(A)** Neurospheres in CTL and rTMS group (*n* = 3). **(B)** Neurosphere diameter increased in rTMS groups (1.24 and 1.58 T; *n* = 3). **(C)** BrdU positive NSPCs in CTL and rTMS groups (*n* = 3). **(D)** BrdU positive rate increased in rTMS groups (1.24 and 1.58 T; *n* = 3). **(E)** Apoptosis rate increased in rTMS groups (1.24 and 1.58 T) assessed by cell death ELISA (*n* = 3). **(F)** Apoptosis rate in CTL and rTMS groups assessed by flow cytometry analysis (*n* = 3). **(G)** Apoptosis rate increased in the rTMS group (1.24 and 1.58 T; *n* = 3). **(H)** Cell viability increased in the rTMS group (1.24 and 1.58 T; *n* = 3). *p < 0.05 compared with the control group. Bar = 100 μm.

The effect of different intensities of rTMS on NSPCs was evaluated at three different values (0.87, 1.24, and 1.58 T), while the frequency and the number of pulses were set to 20 Hz and 1,500, respectively. Our results showed that rTMS (1.24- and 1.58-T groups) stimulated NSPC proliferation (*p* < 0.05), as indicated by the neurosphere diameter (Figures 4A,B) and BrdU positive cell rate (Figures 4C,D). Moreover, the apoptosis rate of NSPCs increased (*p* < 0.05) according to apoptosis ELISA analysis (Figure 4E) and flow cytometry results (Figures 4F,G). CCK-8 assay indicated that intensities of 1.24 and 1.58 T increased cell viability (*p* < 0.05), with the highest values at 1.58-T rTMS intensity (Figure 4H). The intensity at 0.87 T did not show significant differences with respect to the control group. Overall, these results showed that 1.24- and 1.58-T intensities promoted the NSPC proliferation and increase cell viability.

### Different Number of Pulses of rTMS on NSPC Proliferation and Apoptosis

**Figure 5:**
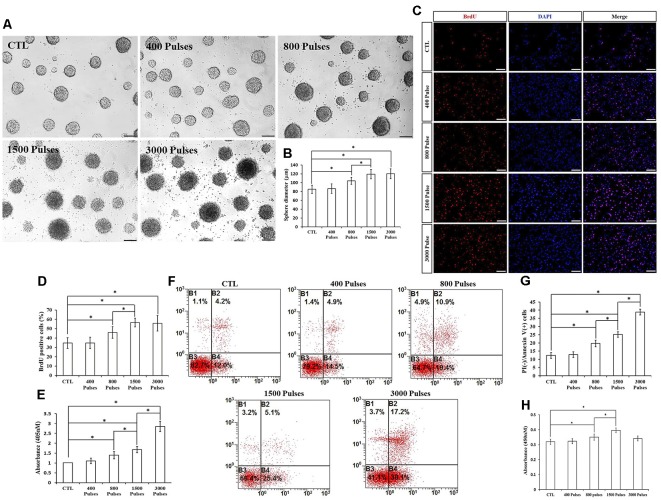
Effects of the different number of pulses of rTMS on NSPC proliferation and apoptosis. **(A)** Neurospheres in CTL and rTMS groups (*n* = 3). **(B)** Neurosphere diameter increased in rTMS groups (800, 1,500, and 3,000 pulses; *n* = 3). **(C)** BrdU positive NSPCs in CTL and rTMS groups (*n* = 3). **(D)** BrdU positive rate increased in rTMS groups (800, 1,500, and 3,000 pulses; *n* = 3). **(E)** Apoptosis rate increased in rTMS groups (800, 1,500, and 3,000 pulses) assessed by cell death ELISA (*n* = 3). **(F)** Apoptosis rate in CTL and rTMS groups assessed by flow cytometry analysis (*n* = 3). **(G)** Apoptosis rate increased in the rTMS group (800, 1,500, and 3,000 pulses; *n* = 3). **(H)** Cell viability increased in the rTMS group (800 and 1,500 pulses; *n* = 3). *p < 0.05 compared with the control group. Bar = 100 μm.

We assayed four different parameters for the number of pulses (400, 800, 1,500, and 3,000) in order to determine whether they affected NSPC proliferation and viability. To do this, we kept the intensity and the frequency set to 1.58 T and 20 Hz, respectively. Compared to the control group, the parameters of 800, 1,500, and 3,000 pulses stimulated NSPC proliferation (*p* < 0.05), but there were no significant differences between the 1,500 and 3,000 groups (*p* > 0.05). Indeed, the neurosphere diameter (Figures 5A,B) and BrdU positive cell rate (Figures 5C,D) clearly showed these effects. Moreover, different pulses significantly increased the NSPC apoptosis rate (*p* < 0.05), as suggested by ELISA data (Figure 5E) and flow cytometry assay (Figures 5F,G). CCK-8 assay showed that 800 and 1,500 pulses increased the cell viability (*p* < 0.05), with the highest value in the 1,500 group (Figure 5H). Due to the rapid increase in the apoptosis rate at 3,000 pulses, the final cell viability had no significant differences with the control group (*p* > 0.05). The pulse number of 400 did not show significant differences with respect to the control group. Taken together, these results indicated that both 800 and 1,500 pulses promote NSPC proliferation and increase cell viability.

### Effects of rTMS on BDNF Signaling in NSPCs

**Figure 6:**
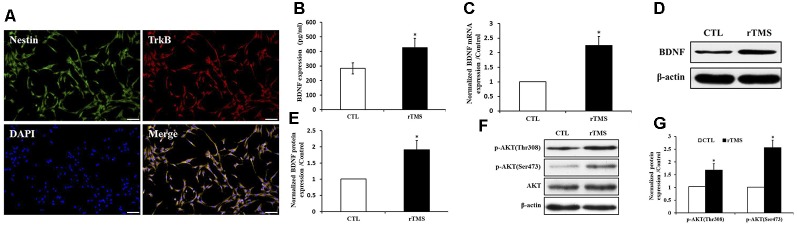
rTMS promoted brain-derived neurotrophic factor (BDNF) expression, which is associated with Akt phosphorylation. **(A)** BDNF receptor TrkB coexpressed with Nestin in NSPCs. **(B)** BDNF secretion was increased in the rTMS group (*n* = 3). **(C)** Normalized *BDNF* mRNA expression was elevated in the rTMS group (*n* = 3). **(D)** BDNF protein expression in NSPCs (*n* = 3). **(E)** Normalized BDNF protein expression was increased in the rTMS group (*n* = 3). **(F)** Expression of phosphorylation of Akt at Thr308, Ser473, and Akt in NSPCs (*n* = 3). **(G)** Normalized expression of phosphorylation of Akt at Thr308 and Ser473 increased in the rTMS group (*n* = 3). *p < 0.05 compared with the control group. Bar = 100 μm.

BDNF receptor TrkB along with Nestin were expressed in NSPCs (Figure 6A). The secretion of BDNF was increased after rTMS stimulation (*p* < 0.05; Figure 6B). Compared to the control group, the *BDNF* expression was significantly upregulated in the rTMS group at the mRNA and protein level (*p* < 0.05 for all; Figures 6C–E). In order to further explore BDNF signaling, we investigated the activation of the Akt pathway. Both p-Akt (Thr308) and p-Akt (Ser473) were significantly upregulated in the rTMS group compared to the control, while no significant differences were observed in total Akt expression (Figures 6F,G). Overall, these results indicated that rTMS stimulation promoted the expression of *BDNF*, which is associated with the phosphorylation level of Akt.

## Discussion

Spinal cord-derived NSPCs are a promising candidate for treating SCI. Transplantation of spinal cord-derived NSPCs in the earlier period after SCI promotes neuron regeneration and function recovery (Sabelström et al., 2014). Unfortunately, how to improve the poor post-transplantation survival and deficiency in differentiation remains a major challenge. Moreover, endogenous NSPCs play a pivotal role in the repair after SCI. rTMS is a treatment used for central nervous system diseases that may achieve satisfactory curative effectiveness. For example, high-frequency rTMS promotes cell proliferation and inhibits apoptosis in oxygen and glucose deprivation/reoxygenation (OGD/R)-injured cells and showed positive results in treating individuals with physical impairments (De Araújo et al., 2017; Baek et al., 2018). Multiple rTMS sessions also have a potential as a treatment for central neuropathic pain (Quesada et al., 2018; Yang et al., 2018), and it also promotes neuron survival and proliferation and reduces the apoptosis rate after traumatic brain injury (Lu et al., 2017). rTMS appeared to be a promising way to promote the transplantation success rate of the transplanted NSPCs and endogenous NSPC survival. In this study, we investigated rTMS stimulation effects on NSPC proliferation and apoptosis upon different conditions, including different intensity, frequency, and number of pulses of magnetic stimulation. We found that 1,500 pulses, 20 Hz, and 1.58 T were the most appropriate parameters.

High-frequency stimulation has been proved to elevate neuroblastoma cells and NSPC proliferation, along with neuronal differentiation *in vitro*, while rTMS treatment at low and high frequencies promoted neuronal differentiation of NSPCs in the subventricular zone of the adult mouse brain (Abbasnia et al., 2015; Lee et al., 2015). In a focal cerebral ischemia rat model, rTMS promoted neural stem cell proliferation *via* the regulation of MiR-25 and the upregulation of MiR-106b *in vitro* (Guo et al., 2014; Liu et al., 2015). However, few studies focused on the effect of rTMS on the spinal cord-derived NSPCs. Previous studies showed that neural stem cells propagated from the adult spinal cord were different from the well-studied forebrain neural stem cells, which maintained neurogenesis throughout the entire organism life (Barnabé-Heider and Frisén, 2008). In this study, NSPCs derived from the adult mouse spinal cord were isolated, cultured, and differentiated under various conditions.

The effect of rTMS depends on the intensity and frequency of the applied stimulation. Depending on the stimulation frequency, the cortical excitability could be temporarily modulated and the physiology within the cortex was consistently regulated by rTMS (Pleger et al., 2006). A previous study showed that low-frequency (1 Hz) rTMS treatment could improve the motor function in cortical stroke patients (Emara et al., 2010). Our previous study revealed that low-frequency rTMS to the contralesional hemisphere combined with high-frequency rTMS to the lesional hemisphere was efficacious in accelerating upper limb motor recovery in patients with acute stroke. In particular, the combination of different-frequencies rTMS was more beneficial for motor improvement than the low-frequency rTMS use only (Long et al., 2018). Herein, we found that rTMS of 1, 10, and 20 Hz significantly increased NSPC proliferation and apoptosis. However, the effect of rTMS on proliferation is more obvious. Therefore, the results of CCK-8 showed that the value of all three experimental groups was higher than that of the control group, but there was no significant difference among the three experimental groups. Although different frequencies of rTMS can lead to different excitability, the effects observed here on NSPC viability were similar.

rTMS intensity also played a decisive role in the therapy of many diseases of the nervous system. rTMS targeting the primary motor cortex (M1) has shown clinical effectiveness in treating patients with neuropathic pain (Ayache et al., 2016). Shimizu et al. (2017) reported that, compared to figure-8 coil, the use of deep rTMS with Hesed coil (H-coil), referred to as “deep TMS,” achieved an improved deeper penetration in the lower limb region of the M1 providing significant short-term pain relief in patients with neuropathic pain. This clinical trial indicated that differences in field intensity, due to different effective depth of rTMS, may be responsible for different clinical outcomes. In this study, the different intensities of rTMS depended on the distances from the center of the coil to the bottom of the culture dish. The higher intensity of rTMS induced higher proliferation and apoptosis in NPSCs. Overall, 1.24 and 1.58 T promoted NSPC proliferation and increased cell viability, indicating that rTMS did not work effectively when the distance from the center of the coil to functional areas is beyond 2 cm, which may be a useful clue in clinical practice.

The number of pulses of rTMS varies in clinical efficacy. Previous studies showed that rTMS treatment consisting of 10 daily sessions of 1,500 pulses over the intact motor cortex could rebalance motor excitability of stroke patients with mild motor injury (Avenanti et al., 2012). Moreover, five daily sessions of 900 pulses over the motor cortex of the influenced hemisphere could accelerate recovery in acute stroke patients (Khedr et al., 2009). However, 200 pulses of rTMS seemed to have a limited effect on improving muscle function and purposeful movement early after stroke, although motor-evoked potential frequency increased 14% for biceps and 20% for triceps after stimulation (Pomeroy et al., 2007). These results suggest that some specific ranges of stimulation pulses may achieve a therapeutic effect. Indeed, we found that 800, 1,500, and 3,000 pulses of rTMS induce NSPC proliferation and apoptosis. Overall, the cell viability upon stimulation with 1,500 pulses induced better results, indicating that a different number of pulses (800, 1,500) could promote NSPC proliferation and increase cell viability. Three thousand pulses of stimulation dramatically increased apoptosis of NSPCs leading to a decrease in the CCK-8 value, and consequently counteracting the proliferative role and finally leading to equivalence of CCK-8 viability to controls.

BDNF, a well-studied neurotrophin, has been proved to be associated with proliferation, neuronal survival, plasticity, and neurotransmitter regulation. BDNF activates its receptor TrkB and leads to intracellular signaling cascades, which include the Ras/ERK protein kinase pathway, the phosphatidylinositol-3-kinase (PI-3 kinase)/Akt kinase pathway, and phospholipase C (PLC)-γ1. Like most members of the AGC kinase family, Akt could be phosphorylated at Thr308 of its T loop and Ser473 of its C-terminal hydrophobic motif (HM) site (Scheid et al., 2002). It has been found that rTMS treatment improves symptoms of refractory depression, especially for agitation, by increasing the plasma level of BDNF (Yukimasa et al., 2006). Low-frequency rTMS also accelerates the motor function recovery in the affected limb after stroke by influencing *BDNF* gene polymorphism (Niimi et al., 2016). Here, we found that the level of BDNF was significantly upregulated in NSPCs after magnetic stimulation, indicating that rTMS promoted BDNF expression and secretion. At the same time, the receptor of BDNF, tropomyosin receptor kinase B (TrkB), was expressed in NSPCs. It has been reported that BDNF/Akt signaling was related to NSPC growth and differentiation *in vitro*, and BDNF secretion as well as the phosphorylation of Akt could be induced by different frequency magnetic stimulation, followed by an influence on neuronal cell proliferation (Sabelström et al., 2014; Hossain et al., 2017; Li et al., 2017). Our results confirmed that the phosphorylation of Akt at Thr308 and Ser473 sites was increased upon magnetic stimulation. Thus, magnetic stimulation might regulate NSPC proliferation correlated to the BDNF/Akt signaling pathway.

In conclusion, we confirmed that some specific parameters of rTMS could promote NSPC proliferation and apoptosis, but the effect on proliferation seemed to be stronger. We identified the most appropriate parameters as 1,500 pulses, 20 Hz, and 1.58 T, useful for further studies on NSPCs *in vitro* and *in vivo*. These effects may be correlated to the BDNF/Akt signaling pathway.

## Data Availability Statement

The raw data supporting the conclusions of this article will be made available by the authors, without undue reservation, to any qualified researcher.

## Ethics Statement

The animal study was reviewed and approved by Medical Ethics Committee of the Xijing Hospital of the Fourth Military Medical University.

## Author Contributions

All authors read, edited, and approved the final manuscript. C-GZ and JQ were responsible for the design and project supervision. WS and FJ were responsible for writing of the manuscript. Y-LZ, RW, and X-LS carried out the experiment. XM and HY are the lead investigators, and developed the design of the study, analysis and interpretations.

## Conflict of Interest

The authors declare that the research was conducted in the absence of any commercial or financial relationships that could be construed as a potential conflict of interest.
